# Supplementary material for: The co-occurrence of genetic variants in the TYR and OCA2 genes confers susceptibility to albinism
Source: Nat Commun. 2024 Sep 30;15:8436. doi: 10.1038/s41467-024-52763-y (PMC11443028; doi:10.1038/s41467-024-52763-y)
Supplement: Supplementary file 3 — Description of Additional Supplementary Files [file 41467_2024_52763_MOESM3_ESM.pdf]

DESCRIPTION OF ADDITIONAL SUPPLEMENTARY FILES

File Name: Supplementary Data 1

Description: Findings in 1,015 probands with albinism who underwent clinical-grade genetic testing at the University Hospital of Bordeaux Molecular Genetics Laboratory.

File Name: Supplementary Data 2

Description: Individuals with albinism from the University Hospital of Bordeaux Molecular Genetics Laboratory (UHB), the Genomics England 100,000 Genomes Project (100K_GP) and the UK Biobank (UKB) datasets who were found to be heterozygous for the *TYR*:c.1205G>A (p.Arg402Gln) and *OCA2*:c.1327G>A (p.Val443Ile) variant combination.

File Name: Supplementary Data 3

Description: Phenotypic information in individuals who were tested for albinism in the University Hospital of Bordeaux Molecular Genetics Laboratory and were found to carry selected genotypes linked to the *TYR*:c.1205G>A (p.Arg402Gln) and/or the *OCA2*:c.1327G>A (p.Val443Ile) variant.

.
