# Supplementary material for: The co-occurrence of genetic variants in the TYR and OCA2 genes confers susceptibility to albinism
Source: Nat Commun. 2024 Sep 30;15:8436. doi: 10.1038/s41467-024-52763-y (PMC11443028; doi:10.1038/s41467-024-52763-y)
Supplement: Supplementary file 5 — Reporting Summary [file 41467_2024_52763_MOESM5_ESM.pdf]

nature portfolio

Corresponding author(s): Panagiotis Sergouniotis

Last updated by author(s): Aug 12, 2024

Reporting Summary

Nature Portfolio wishes to improve the reproducibility of the work that we publish. This form provides structure for consistency and transparency in reporting. For further information on Nature Portfolio policies, see our Editorial Policies and the Editorial Policy Checklist.

Statistics

For all statistical analyses, confirm that the following items are present in the figure legend, table legend, main text, or Methods section.

n/a	Confirmed	
☐	☒	The exact sample size (*n*) for each experimental group/condition, given as a discrete number and unit of measurement
☒	☐	A statement on whether measurements were taken from distinct samples or whether the same sample was measured repeatedly
☐	☒	The statistical test(s) used AND whether they are one- or two-sided<br>*Only common tests should be described solely by name; describe more complex techniques in the Methods section.*
☐	☒	A description of all covariates tested
☐	☒	A description of any assumptions or corrections, such as tests of normality and adjustment for multiple comparisons
☐	☒	A full description of the statistical parameters including central tendency (e.g. means) or other basic estimates (e.g. regression coefficient) AND variation (e.g. standard deviation) or associated estimates of uncertainty (e.g. confidence intervals)
☐	☒	For null hypothesis testing, the test statistic (e.g. *F*, *t*, *r*) with confidence intervals, effect sizes, degrees of freedom and *P* value noted<br>*Give P values as exact values whenever suitable.*
☒	☐	For Bayesian analysis, information on the choice of priors and Markov chain Monte Carlo settings
☒	☐	For hierarchical and complex designs, identification of the appropriate level for tests and full reporting of outcomes
☒	☐	Estimates of effect sizes (e.g. Cohen's *d*, Pearson's *r*), indicating how they were calculated

Our web collection on statistics for biologists contains articles on many of the points above.

Software and code

Policy information about availability of computer code

Data collection	No specific software was used for data collection.
Data analysis	BCFtools v1.10.2; Plink v1.9; vcftools 0.1.15; logistf v1.24.1; Graphpad Prism v9; Python v3, R v4.0.4, basic scripts for data processing and running the previously mentioned tools can be found at https://github.com/davidjohngreen/tyr-oca2

For manuscripts utilizing custom algorithms or software that are central to the research but not yet described in published literature, software must be made available to editors and reviewers. We strongly encourage code deposition in a community repository (e.g. GitHub). See the Nature Portfolio guidelines for submitting code & software for further information.

Data

Policy information about availability of data

All manuscripts must include a data availability statement. This statement should provide the following information, where applicable:

- Accession codes, unique identifiers, or web links for publicly available datasets
- A description of any restrictions on data availability
- For clinical datasets or third party data, please ensure that the statement adheres to our policy

Genomics England 100,000 Genomes Project data are available under restricted access through a procedure described at https://www.genomicsengland.co.uk/about-gecip/for-gecip-members/data-and-data-access. UK Biobank data are available under restricted access through a procedure described at http://

www.ukbiobank.ac.uk/using-the-resource/. All other data supporting the findings of this study are available within the article (including its supplementary information files) or from the authors upon request.

Research involving human participants, their data, or biological material

Policy information about studies with human participants or human data. See also policy information about sex, gender (identity/presentation), and sexual orientation and race, ethnicity and racism.

Reporting on sex and gender	Information on the biological sex of individuals from the University Hospitals of Bordeaux albinism cohort, the Genomics England 100,000 Genomes Project cohort, and the UK Biobank dataset was processed and this parameter was included as a covariate in some of the analyses (where stated).
Reporting on race, ethnicity, or other socially relevant groupings	In the case of UK Biobank and Genomics England data, ancestry was inferred from the principle component projections (provided in the relevant resources).<br><br>For the 1,120 individuals from the University Hospitals of Bordeaux, data on ancestry were self-reported and categorized into broad ancestral groups manually.
Population characteristics	1,120 individuals from the University Hospitals of Bordeaux albinism cohort plus 29,451 individuals from the Genomics England 100,000 Genomes Project were included in this study; data from 131,985 individuals from the UK Biobank (who had enhanced ophthalmic phenotyping) were also inspected.<br><br>Of the 1,120 individuals from the University Hospitals of Bordeaux, 44% were female and 84% were of European Ancestries.<br><br>Most study subjects were of European ancestries. Self-reported ancestry data were collected from individuals from the University Hospitals of Bordeaux albinism cohort. For the Genomics England 100,000 Genomes Project and the UK Biobank datasets, ancestry was determined using the PCA projections provided in these resources.<br><br>In terms of diagnosis (and assignment to the case or the control group), for the University Hospitals of Bordeaux cohort (reported in Supplementary Dataset 1), albinism was diagnosed via clinical assessment and gene panel testing. For the Genomics England 100,000 Genomes Project and the UK Biobank datasets, the clinical (albinism) status was determined after inspection of the relevant information provided in these resources (including the assigned HPO/ICD codes).
Recruitment	Individuals in the University Hospitals of Bordeaux albinism cohort were recruited through the database of the University Hospital of Bordeaux Molecular Genetics Laboratory. This is a national reference laboratory that has been performing genetic testing for albinism since 2003 and has been receiving samples from individuals predominantly based in France (or French-administered overseas territories).<br><br>Individuals in the Genomics England 100,000 Genomes Project were recruited as part of a national genome sequencing initiative. Enrollment was coordinated by Genomics England Limited, and participants were recruited mainly at National Health Service (NHS) Hospitals in the UK.<br><br>The UK Biobank is a biomedical resource containing genetic and health information from >500,000 individuals from across the UK (aged 40–70 years at baseline). The UK Biobank, invited approximately 9 million UK residents, obtaining a 6% response rate of people who volunteered and became participants, a 6% that is known to be characterized by healthy volunteer bias
Ethics oversight	The study of individuals from the University Hospital of Bordeaux albinism cohort has been approved by the relevant local ethics committee (Comité de Protection des Personnes Sud-Ouest et Outre Mer III, Bordeaux, France).<br><br>The informed consent process for the Genomics England 100,000 Genomes Project has been approved by the National Research Ethics Service Research Ethics Committee for East of England – Cambridge South Research Ethics Committee.<br><br>The UK Biobank has received approval from the National Information Governance Board for Health and Social Care and the National Health Service North West Centre for Research Ethics Committee (Ref: 11/NW/0382).<br><br>All investigations were conducted in accordance with the tenets of the Declaration of Helsinki.

Note that full information on the approval of the study protocol must also be provided in the manuscript.

Field-specific reporting

Please select the one below that is the best fit for your research. If you are not sure, read the appropriate sections before making your selection.

☒ Life sciences ☐ Behavioural & social sciences ☐ Ecological, evolutionary & environmental sciences

For a reference copy of the document with all sections, see nature.com/documents/nr-reporting-summary-flat.pdf

Life sciences study design

All studies must disclose on these points even when the disclosure is negative.

Sample size	No sample size calculation was performed.
Data exclusions	For the UK Biobank data, redacted individuals were excluded. For the Genomics England 100,000 Genomes data, individuals who had withdrawn from the study at the time of analysis were removed.
Replication	Not applicable
Randomization	Not applicable
Blinding	Not applicable as no treatment or intervention was investigated.<br>This study is a retrospective analysis of data collected by hospital services and by the Genomics England and the UK Biobank projects.

Reporting for specific materials, systems and methods

We require information from authors about some types of materials, experimental systems and methods used in many studies. Here, indicate whether each material, system or method listed is relevant to your study. If you are not sure if a list item applies to your research, read the appropriate section before selecting a response.

Materials & experimental systems

n/a	Involved in the study	
☒	☐	Antibodies
☒	☐	Eukaryotic cell lines
☒	☐	Palaeontology and archaeology
☒	☐	Animals and other organisms
☐	☒	Clinical data
☒	☐	Dual use research of concern
☒	☐	Plants

Methods

n/a	Involved in the study	
☒	☐	ChIP-seq
☒	☐	Flow cytometry
☒	☐	MRI-based neuroimaging

Clinical data

Policy information about clinical studies

All manuscripts should comply with the ICMJE guidelines for publication of clinical research and a completed CONSORT checklist must be included with all submissions.

Clinical trial registration	This is an observational study (not a clinical trial).
Study protocol	*Note where the full trial protocol can be accessed OR if not available, explain why.*
Data collection	*Describe the settings and locales of data collection, noting the time periods of recruitment and data collection.*
Outcomes	*Describe how you pre-defined primary and secondary outcome measures and how you assessed these measures.*

Plants

Seed stocks	Not applicable
Novel plant genotypes	Not applicable
Authentication	Not applicable
